# Interplay of Klebsiella pneumoniae *fabZ* and *lpxC* Mutations Leads to LpxC Inhibitor-Dependent Growth Resulting from Loss of Membrane Homeostasis

**DOI:** 10.1128/mSphere.00508-18

**Published:** 2018-10-31

**Authors:** Mina Mostafavi, Lisha Wang, Lili Xie, Kenneth T. Takeoka, Daryl L. Richie, Fergal Casey, Alexey Ruzin, William S. Sawyer, Christopher M. Rath, Jun-Rong Wei, Charles R. Dean

**Affiliations:** a Infectious Diseases, Novartis Institutes for BioMedical Research, Emeryville, California, USA; University of Rochester

**Keywords:** LpxC, *fabZ*, lipid A, toxic accumulation

## Abstract

Tight coordination of inner and outer membrane biosynthesis is very important in Gram-negative bacteria. Biosynthesis of the lipid A moiety of lipopolysaccharide, which comprises the outer leaflet of the outer membrane has garnered interest for Gram-negative antibacterial discovery. In particular, several potent inhibitors of LpxC (the first committed step of the lipid A pathway) are described. Here we show that serial passaging of Klebsiella pneumoniae in increasing levels of an LpxC inhibitor yielded mutants that grew only in the presence of the inhibitor. These strains had mutations in *fabZ* and *lpxC* occurring together (encoding either FabZ_R121L_/LpxC_V37G_ or FabZ_F51L_/LpxC_V37G_). K. pneumoniae mutants having only LpxC_V37G_ or LpxC_V37A_ or various FabZ mutations alone were less susceptible to the LpxC inhibitor and did not require LpxC inhibition for growth. Western blotting revealed that LpxC_V37G_ accumulated to high levels, and electron microscopy of cells harboring FabZ_R121L_/LpxC_V37G_ indicated an extreme accumulation of membrane in the periplasm when cells were subcultured without LpxC inhibitor. Significant accumulation of detergent-like lipid A pathway intermediates that occur downstream of LpxC (e.g., lipid X and disaccharide monophosphate [DSMP]) was also seen. Taken together, our results suggest that redirection of lipid A pathway substrate by less active FabZ variants, combined with increased activity from LpxC_V37G_ was overdriving the lipid A pathway, necessitating LpxC chemical inhibition, since native cellular maintenance of membrane homeostasis was no longer functioning.

**IMPORTANCE** Emergence of antibiotic resistance has prompted efforts to identify and optimize novel inhibitors of antibacterial targets such as LpxC. This enzyme catalyzes the first committed step of lipid A synthesis, which is necessary to generate lipopolysaccharide and ultimately the Gram-negative protective outer membrane. Investigation of this pathway and its interrelationship with inner membrane (phospholipid) biosynthesis or other pathways is therefore highly important to the fundamental understanding of Gram-negative bacteria and by extension to antibiotic discovery. Here we exploited the availability of a novel LpxC inhibitor to engender the generation of K. pneumoniae resistant mutants whose growth depends on chemical inhibition of LpxC. Inhibitor dependency resulted from the interaction of different resistance mutations and was based on loss of normal cellular mechanisms required to establish membrane homeostasis. This study provides new insights into the importance of this process in K. pneumoniae and how it may be linked to novel biosynthetic pathway inhibitors.

## Abstract (precis)

Emergence of antibiotic resistance has prompted efforts to identify and optimize novel inhibitors of antibacterial targets such as LpxC. This enzyme catalyzes the first committed step of lipid A synthesis, which is necessary to generate lipopolysaccharide and ultimately the Gram-negative protective outer membrane. Investigation of this pathway and its interrelationship with inner membrane (phospholipid) biosynthesis or other pathways is therefore highly important to the fundamental understanding of Gram-negative bacteria and by extension to antibiotic discovery. Here we exploited the availability of a novel LpxC inhibitor to engender the generation of K. pneumoniae resistant mutants whose growth depends on chemical inhibition of LpxC. Inhibitor dependency resulted from the interaction of different resistance mutations and was based on loss of normal cellular mechanisms required to establish membrane homeostasis. This study provides new insights into the importance of this process in K. pneumoniae and how it may be linked to novel biosynthetic pathway inhibitors.

## INTRODUCTION

The emergence of antibiotic resistance is increasingly recognized as a serious public health threat (1, 2) and has generated urgent calls for the development of new therapies for the treatment of infections caused by multidrug-resistant pathogens (2, 3). An area of strong interest as a potential target for antibacterial discovery vis-a-vis Gram-negative pathogens is the biosynthetic pathway for the lipid A component of lipopolysaccharide (LPS) (4), which is necessary for the formation of the Gram-negative outer membrane (OM). Lipid A comprises the outer leaflet of the OM, with phospholipid forming the inner leaflet (5). Lipid A is decorated with a core oligosaccharide and may also have repeating O-antigen polysaccharide units extending out from the cell surface. LPS (lipid A) is essential for the growth of many important pathogens, such as Escherichia coli, Klebsiella pneumoniae, and Pseudomonas aeruginosa, although other pathogens can survive without LPS (e.g., some Acinetobacter baumannii [6–9] and Neisseria meningitidis [10]). The OM also provides an often significant permeability barrier to toxic compounds, including many antibiotics, and also protects the cells from host immune components such as serum complement (11, 12).

**FIG 1:**
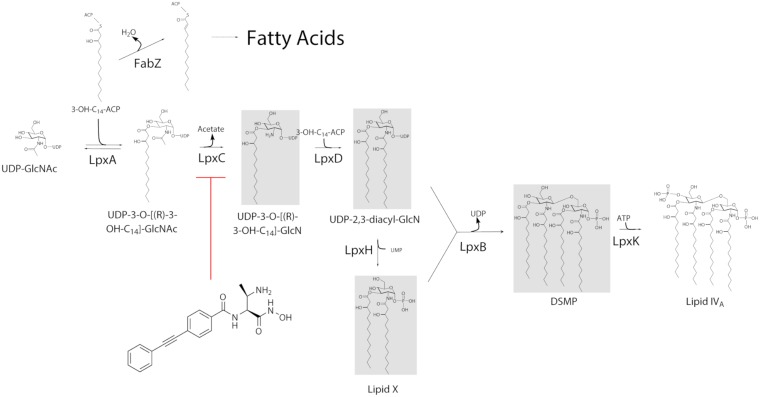
Biochemical pathway of *E. coli* lipid A biosynthesis up to lipid IVA. Involvement of the common precursor (3-OH-C14-ACP) in the synthesis of lipid A and saturated fatty acids is indicated. The structure of LpxC inhibitor compound 2 (14) is shown. UDP-GlcNAc, uridine diphosphate (UDP)-*N*-acetylglycosamine; ACP, acyl carrier protein.

The first reaction of the lipid A biosynthetic pathway (Fig. 1), catalyzed by LpxA, is acylation of UDP-GlcNAc by transfer of the *R*-3-hydroxyacyl chain from *R*-3-hydroxyacyl-acyl carrier protein (ACP). *R*-3-Hydroxyacyl-ACP is also a substrate of FabZ in the fatty acid biosynthetic pathway (Fig. 1). LpxC, a zinc-dependent deacetylase, then catalyzes the formation of UDP-3-*O*-(*R*-3-hydroxyacyl)-GlcN, which feeds into LpxD and later steps of the pathway. LpxC is the first committed step in the lipid A biosynthetic pathway, and as a zinc-dependent enzyme, lends itself to the development of hydroxamate-warhead-based inhibitors (13–15). Therefore, the disruption of LPS, either directly by compounds such as polymyxins or indirectly via inhibition of biosynthetic targets such as LpxC should in some instances increase susceptibility to other antibiotics (16) or decrease survival in the host (17). Supporting the latter, an inhibitor of LpxC could cure A. baumannii infection in an animal model despite its lack of *in vitro* antibacterial activity (17). Because of these factors, LpxC is an extensively explored target in the LPS pathway and several potent inhibitors have been described (18–34), although to date, none have completed phase I clinical trials.

Several mechanisms decreasing *in vitro* susceptibility to LpxC inhibitors have been identified in different organisms (35–37). These mechanisms include upregulation of active efflux, overexpression or alteration of the target protein LpxC (35), or mutations in fatty acid biosynthetic genes (*fabG* in P. aeruginosa or *fabZ* in E. coli and K. pneumoniae) (15, 35–39). E. coli *fabZ* mutations were shown to encode FabZ proteins with reduced activity (37, 40), which has been proposed to elevate the distribution of *R*-3-hydroxyacyl-ACP substrates into the lipid A biosynthetic pathway (Fig. 1) which ultimately competes with the LpxC inhibitor compound for binding to LpxC (36). Studies of E. coli *fabZ* mutants in the absence of exposure to an LpxC inhibitor showed that the cells reduce cellular levels of LpxC to balance fatty acid and lipid A synthesis in order to restore membrane homeostasis (37, 40). Since a key control point of this process was LpxC, it stands to reason that continuous chemical inhibition of LpxC might select mutants where normal biological control of lipid A biosynthesis at the LpxC step was lost. Without LpxC inhibitor, normal membrane homeostasis would be lost, leading to inhibitor-dependent growth. The availability of potent LpxC inhibitors allowed for selection of mutants in the important Gram-negative pathogen K. pneumoniae by serial passaging (14) that, in addition to becoming less susceptible to the inhibitor, indeed became dependent on the inhibitor for growth. This resulted from a combination of *fabZ* and *lpxC* mutations, with the latter appearing to strongly increase cellular levels of altered LpxC protein. The resulting uncontrolled production of lipid A led to an inability to grow unless LpxC was chemically inhibited. The implications of this are discussed.

## RESULTS

### K. pneumoniae LpxC mutants adapted to grow only in the presence of an LpxC inhibitor emerge during serial passaging.

**FIG 2:**
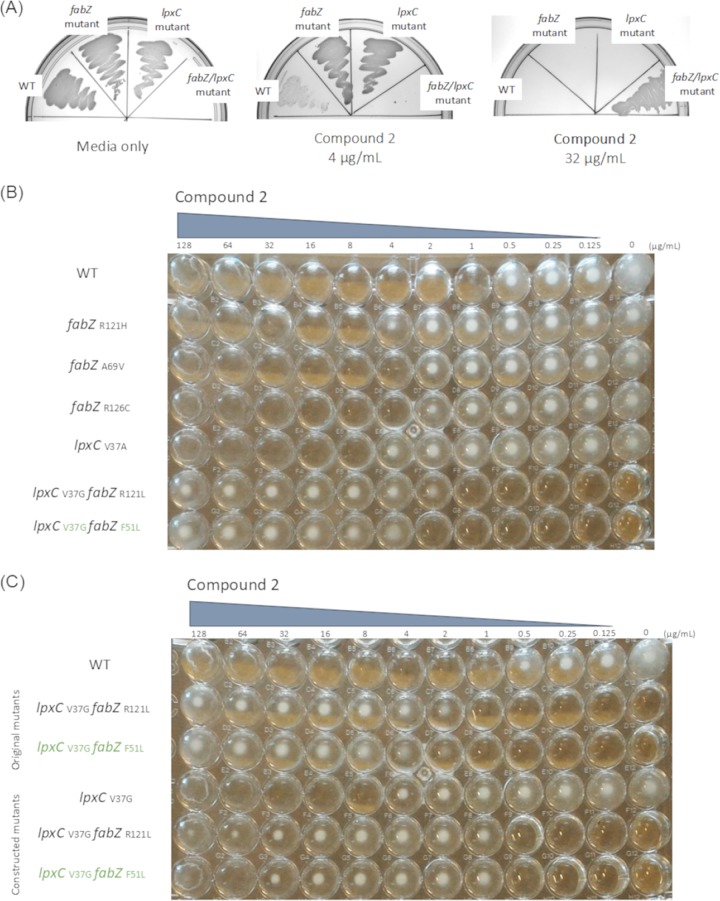
Compound 2-dependent growth of *K. pneumoniae* mutants. (A) *K. pneumoniae* ATCC 43816 (wild type [WT]), *fabZ* mutant (JWM0009 [FabZR121H]), *lpxC* mutant (JWM0107 [LpxCV37A]), or the *fabZ/lpxC* double mutant (JWM0012 [LpxCV37G/FabZR121L]) was streaked on LB agar with compound 2 at 0, 4, or 32 μg/ml. The *fabZ/lpxC* double mutant (JWM0012) grew only in medium containing 32 μg/ml compound 2. (B) *K. pneumoniae* ATCC 43816 (WT), *fabZ* mutants (JWM0009 encoding FabZR121H, JWM0010 encoding FabZA69V, JWM0011 encoding FabZR126C), an *lpxC* mutant (JWM0107 encoding LpxCV37A) or *fabZ/lpxC* double mutants (JWM0012 encoding LpxCV37G/FabZR121L and JWM0013 encoding LpxCV37G/FabZF51L) were assessed for growth in the presence of compound 2 using broth medium. Individual *fabZ* or *lpxC* mutations decreased susceptibility to compound 2 by about four- to eightfold as expected. Both mutations together further enhanced resistance and led to compound 2-dependent growth. (C) The roles of *lpxC* mutation and the combined *lpxC*/*fabZ* mutations were confirmed using engineered *K. pneumoniae* ATCC 43816 derivatives (JWK0148 encoding LpxCV37G, JWK0150 encoding LpxCV37G/FabZR121L, and JWK0151 encoding LpxCV37G/FabZF51L).

Consistent with previous reports (15, 36, 37), single-step mutant selection using various LpxC inhibitors consistently yielded *fabZ* mutants (e.g., strains JWM0009 encoding FabZ_R121H_, JWM0010 encoding FabZ_A69V_, and JWM0011 encoding FabZ_R126C_) and occasionally selected mutations in *lpxC* (JWM0107 encoding LpxC_V37A_) (Fig. 2A and B). The mutants did not exhibit noticeable growth defects on common media (e.g., Mueller-Hinton agar [MHA] or LB agar) lacking the LpxC inhibitor. We surmised that more complex phenomena relating to both reduced susceptibility and pathway interactions would be revealed by a serial passaging approach in the presence of compound 2 to allow for the accumulation of mutations and adaptation to continuous exposure to an LpxC inhibitor. Two mutants recovered from that approach had substantially decreased susceptibility to compound 2 (MIC shifted from 2 to >128 µg/ml). Intriguingly, although resistant to compound 2, these mutants failed to grow in the absence of compound 2 (strains JWM0012 and JWM0013 [Fig. 2A and B]). Additional LpxC inhibitors such as CHIR-090 (31) also induced growth of strains JWM0012 and JWM0013 (data not shown); therefore, the effect was not specific to the compound used for selection.

### LpxC inhibitor-dependent growth of K. pneumoniae mutants JWM0012 and JWM0013 is mediated by the combined effect of mutations in *fabZ* and *lpxC*.

Whole-genome sequencing of K. pneumoniae JWM0012 and JWM0013 revealed the presence of mutations in *lpxC* and *fabZ* (encoding LpxC_V37G_/FabZ_R121L_ and LpxC_V37G_/FabZ_F51L_, respectively) along with additional mutations (see Table S1 in the supplemental material). Since *lpxC* and *fabZ* mutations were in common between these two mutants, these were both engineered into the susceptible parent K. pneumoniae ATCC 43816 strain background using recombineering. Mutant JWK0148 encoding only the LpxC_V37G_ mutation showed decreased susceptibility to compound 2, indicating that this LpxC variant is itself a determinant of *in vitro* resistance to an LpxC inhibitor (Fig. 2C). JWK0148 growth was not dependent on compound 2. To our knowledge, this and the LpxC_V37A_ variant of JWM0107 isolated via single-step selection are the first descriptions of LpxC target alterations reducing susceptibility to an LpxC inhibitor in K. pneumoniae.

10.1128/mSphere.00508-18.4 TABLE S1 Strains and plasmids used in this study. Download Table S1, PDF file, 0.1 MB. Copyright © 2018 Mostafavi et al. 2018 Mostafavi et al. This content is distributed under the terms of the Creative Commons Attribution 4.0 International license.

Mutants JWK0150 and JWK0151 were engineered to encode LpxC_V37G_ plus FabZ_R121L_ or FabZ_F51L,_ respectively. Each of these engineered mutants required compound 2 in order to grow (Fig. 2C). It should be noted that the originally selected mutants required somewhat higher levels of compound 2 to induce growth than the engineered mutants and were ultimately even less susceptible to the inhibitor (Fig. 2C). This likely resulted from the presence of additional mutations in the passaged mutants contributing to decreasing susceptibility to compound 2. Indeed, isolated mutant JWM0012 has an additional mutation inactivating RamR (Table S1). RamR is the transcriptional repressor of RamA, which is a transcriptional activator of efflux pumps AcrAB (41). Recently, RamA was also reported to activate transcription of genes in the lipid A biosynthesis pathway, including *lpxC* (42).

### LpxC_V37G_ and LpxC_G36R_ accumulate to high levels in the cell.

We mapped the selected LpxC mutations onto an available E. coli LpxC inhibitor costructure. K. pneumoniae LpxC_V37G,_ as well as LpxC_G36R_ and LpxC_V37G_ variants that we had selected for reduced susceptibility to the LpxC inhibitor in E. coli (Table S1), localized at a distance from the binding pocket (see Fig. S1 in the supplemental material). A previously described E. coli variant LpxC_I38T_, which decreases susceptibility to an LpxC inhibitor BB-78484 (36) that is structurally different from compound 2, also localized to this region of the protein. These results suggested that this region of LpxC is important for determining susceptibility to a range of different LpxC inhibitors.

10.1128/mSphere.00508-18.1 FIG S1 Locations of amino acid substitutions in E. coli LpxC that reduce susceptibility to LpxC inhibitors, based on E. coli LpxC in complex with LPC-009 (PDB accession no. 3P3G [C. J. Lee, X. Liang, X. Chen, D. Zeng, et al., Chem Biol **18:**38‐47, 2011]). LPC-009 is shown with a stick model, and the active site zinc ion is shown with a space-filling model. The previously reported substitutions at I38 and two identified here at G36 and V37 are distant from the ligand binding site. Changes at residues highlighted in purple suppress the effect of V37G and allow growth of cells in the absence of compound 2. Download FIG S1, PDF file, 0.2 MB. Copyright © 2018 Mostafavi et al. 2018 Mostafavi et al. This content is distributed under the terms of the Creative Commons Attribution 4.0 International license.

**FIG 3:**
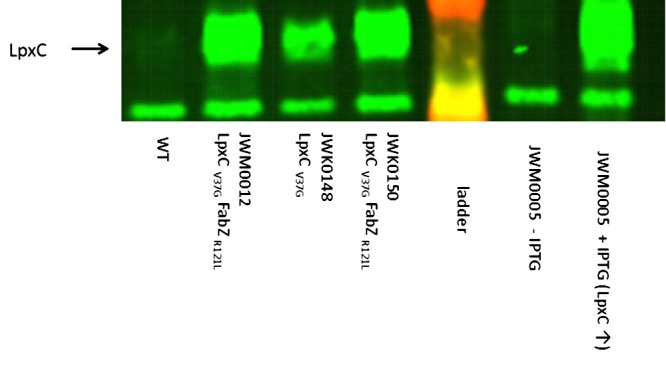
Cellular levels of LpxCV37G assessed by Western blotting. Cultures were collected when grown in exponential phase with compound 2 at 32 μg/ml. From left to right, lane 1, level of wild-type LpxC in *K. pneumoniae* ATCC 43816; lane 2, level of LpxCV37G in the passaged *fabZ*/*lpxC* mutant JWM0012; lane 3, level of LpxCV37G in the single *lpxC* mutant JWK0148; lane 4, level of LpxCV37G in the reconstructed *fabZ/lpxC* double mutant JWM0150; lane 5, ladder; lanes 6 and 7, control strain JWM0005 with a IPTG-inducible LpxC (uninduced) and induced, respectively. LpxCV37G clearly accumulates to higher levels in cells than wild-type LpxC does (compare lane 3 to lane 1) in the presence or absence of compound 2. LpxCV37G appears to be even more abundant in the *fabZ*/*lpxC* double mutants (compare lanes 2 and 4 to lane 1). The position of LpxC is indicated by the black arrow to the left of the gel, and an extra nonspecific band was shown as a control for loading.

Since these mutations did not appear to affect the LpxC inhibitor binding site, we hypothesized that they might act by somehow mediating increased LpxC protein levels. Increased abundance of LpxC would be consistent with the ability of these mutations to reduce susceptibility to LpxC inhibitors in general. Western blot analysis showed a clear increase in LpxC abundance relative to the parent strain in K. pneumoniae encoding the LpxC variant LpxC_V37G_ (strain JWM0148) and perhaps even larger accumulation in mutants encoding FabZ_R121L_/LpxC_V37G_ (JWM0012) and FabZ_R121_/LpxC_V37G_ (JWM0150) (Fig. 3). There was a modest (threefold) increased abundance of *lpxC* transcripts in mutants JWK0148 and JWK0150 (data not shown), which presumably contributes to the increased level of LpxC, but the increase of protein abundance is much higher than threefold. This suggests that LpxC_V37G_ may be more stable than wild-type LpxC. Since the V37G substitution is not localized in the compound binding site and may not significantly impact compound 2 binding, increased abundance of LpxC_V37G_ may decrease susceptibility to compound 2 via a compound titration effect.

### FabZ_R121L_ and LpxC_V37G_ together lead to loss of control of the lipid A biosynthetic pathway and severe overproduction of LPS.

**FIG 4:**
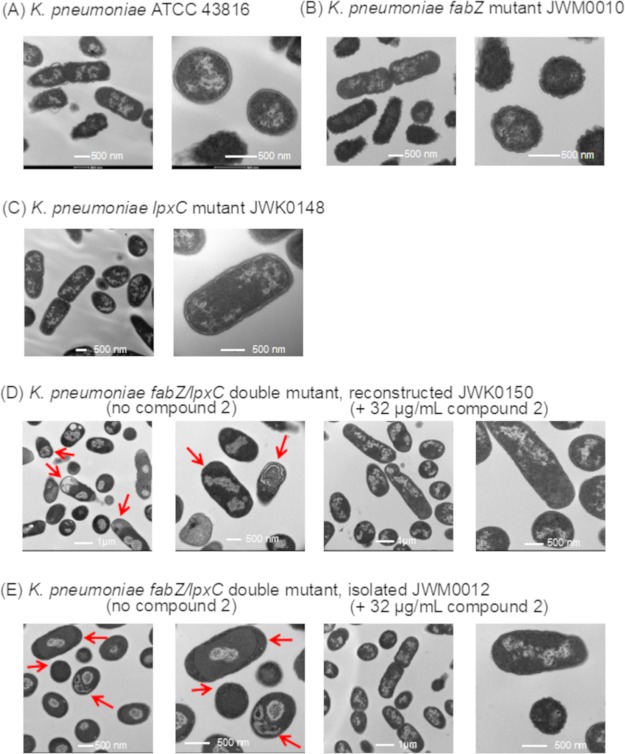
Morphology of *K. pneumoniae* ATCC 43816 (A), *fabZ* mutant (JWM0010) (B), *lpxC* mutant (JWK0148) (C), the reconstructed *fabZ/lpxC* double mutant (JWK0150) (D), and the passaged *fabZ/lpxC* double mutant (JWM0012) (E) as examined by transmission electron microscopy. Compound 2-dependent mutants JWK0150 and JWM0012 exhibited a severe accumulation of membranous material (arrows) when subcultured from medium with compound 2 to medium without compound 2 (left panels). This did not occur in cells subcultured into compound 2-containing medium (right panels). The scale is indicated in each image.

Synthesis of LPS needs to be tightly controlled and balanced with phospholipid (inner membrane) biosynthesis (37, 38). It was recently shown that E. coli responds to the fatty acid pathway defect engendered by *fabZ* mutations by reducing cellular levels of LpxC, presumably decreasing the production of lipid A (37, 43, 44). However, as shown above, LpxC_V37G_ accumulated to levels higher than wild-type levels within cells regardless of the presence or absence of a *fabZ* mutation, and indeed may have accumulated slightly higher levels in the presence of *fabZ* mutations (Fig. 3A and B). Assuming that wild-type K. pneumoniae exhibits similar coregulation of FabZ and LpxC to that described for E. coli, our observations suggest that LpxC_V37G_ interferes with the normal ability to control LpxC levels in response to defects in FabZ. Consistent with this, JWM0012 (FabZ_R121L_/LpxC_V37G_) cells exhibited an extreme accumulation of LPS within the periplasm when subcultured into medium lacking compound 2 (Fig. 4 and Fig. S2) as would be expected from such dysregulation. Similar results were seen for the engineered *fabZ*/*lpxC* mutant JWK0150 (Fig. 4). Interestingly, there were no obvious membrane defects in cells expressing only LpxC_V37G_ alone (Fig. 4C), indicating that increased LpxC levels in this mutant, in the absence of additional substrate redirection resulting from defective FabZ, were insufficient to cause serious LPS accumulations. The extreme level of LPS (especially relative to inner membrane) in the *fabZ*/*lpxC* mutant indicated an inability to establish or maintain membrane homeostasis in these mutants in the absence of compound 2. Compound 2 at 32 µg/ml prevented LPS accumulation (Fig. 4), and exposure to compound 2 also allowed these mutants to grow on MacConkey agar plates that contain bile salts which prevent the growth of Enterobacteriaceae if they have membrane permeability defects (data not shown). Moreover, compound 2 at sublethal levels strongly sensitized wild type, but not the *fabZ/lpxC* mutant K. pneumoniae to rifampin. Therefore, inhibition of LpxC induced an outer membrane permeability defect in wild-type cells, whereas it prevented a membrane defect in the mutants (Fig. S3). The apparent lack of membrane defects in the mutants treated with compound 2 provides further evidence that membrane homeostasis has been restored in the *fabZ*/*lpxC* mutants via chemical inhibition of LpxC.

10.1128/mSphere.00508-18.2 FIG S2 TEM images of K. pneumoniae JWK0012 (isolated *fabZ/lpxC* double mutant) grown overnight in medium supplemented with compound 2 and then subcultured into fresh medium without compound 2 as described in Materials and Methods. Compound 2-dependent mutant JWM0012 exhibited a severe accumulation of membranous material (arrows) when subcultured from medium with compound 2 to medium without compound 2. Download FIG S2, PDF file, 0.2 MB. Copyright © 2018 Mostafavi et al. 2018 Mostafavi et al. This content is distributed under the terms of the Creative Commons Attribution 4.0 International license.

10.1128/mSphere.00508-18.3 FIG S3 Susceptibility of K. pneumoniae ATCC 43816 or the *fabZ/lpxC* double mutants JWM0012 and JWM0013 to compound 2 in the presence or absence of rifampicin (RIF) at 1 μg/ml. In the absence of RIF, the MIC of compound 2 for K. pneumoniae ATCC 43816 is 2 μg/ml. In the presence of RIF, this decreased to ≤0.125 μg/ml, likely reflecting disruption of the bacterial membrane permeability barrier. In contrast, the MIC of compounds for JWM0012 or JWM0013 in the presence of RIF was >128 μg/ml, indicating that the cell envelope permeability barrier is intact. Download FIG S3, PDF file, 0.1 MB. Copyright © 2018 Mostafavi et al. 2018 Mostafavi et al. This content is distributed under the terms of the Creative Commons Attribution 4.0 International license.

### Overproduction of lipid A leads to accumulation of lipid A pathway intermediates.

**FIG 5:**
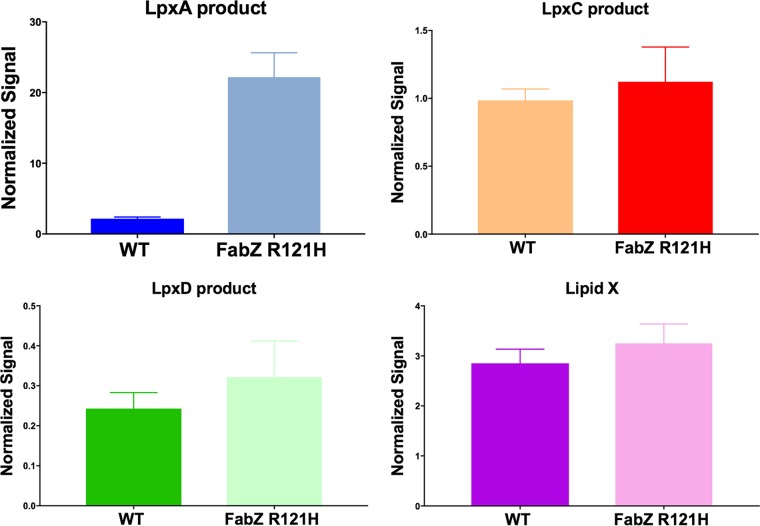
Accumulation of the lipid A pathway intermediate UDP-3-*O*-[(R)-3-OH-C14]-GlcNAc in the *fabZ* (FabZR121H) mutant JWM0009 determined by LC-MS/MS. The increase of β-hydroxymyristoyl-ACP, a precursor molecule for lipid A biosynthesis (Fig. 1), was observed in the *fabZ* mutant, and this finding suggests that FabZR121H is redirecting intermediate flux into the lipid A biosynthetic pathway. As expected, UDP-3-*O*-[(R)-3-OH-C14]-GlcNAc did not accumulate in the *lpxC* mutant JWM0010 (data not shown). Data were normalized to an internal standard (IS) and the culture OD600. Each experiment was averaged from eight replicates.

**FIG 6:**
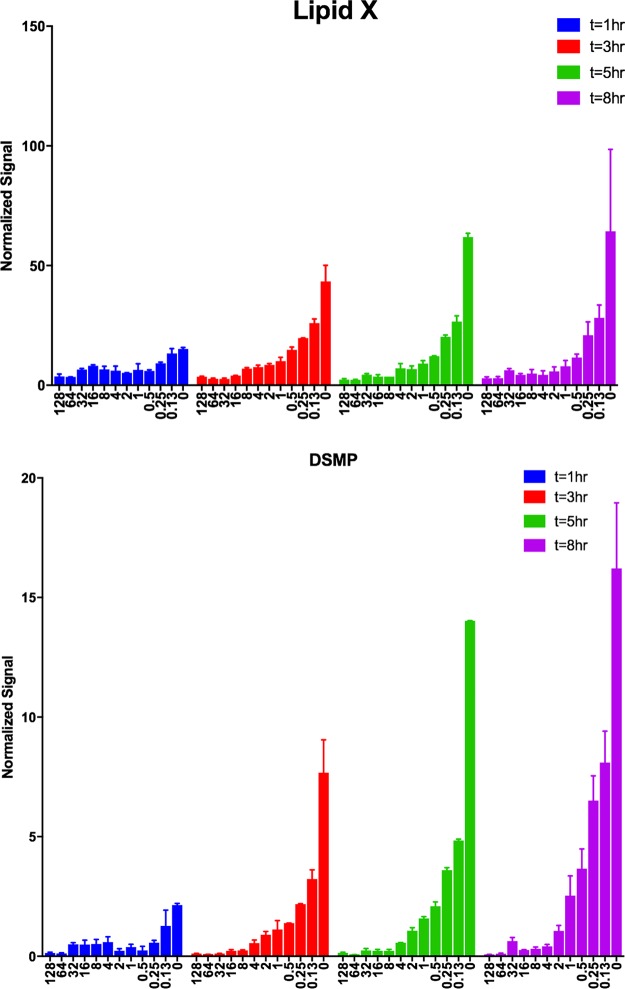
Accumulation of lipid A pathway intermediates lipid X and DSMP in the compound 2-dependent *K. pneumoniae fabZ/lpxC* double mutant JWM0012 when subcultured from medium containing 32 μg/ml compound 2 to medium containing progressively lower levels of the compound. Cells were collected at 1-, 3-, 5-, and 8-h time points for determination of intermediate levels using LC-MS/MS methodology. The concentrations of compound 2 for each sample are indicated on the *x* axes. Normalized signals were plotted on the *y* axes.

The periplasmic accumulation of LPS seen here for our *fabZ*/*lpxC* mutants is reminiscent of that observed in cells with defects in the Lpt LPS transport machinery (45, 46) or in cells treated with the LptD inhibitor POL7080 (47). Therefore, the rate of lipid A (LPS) synthesis was so high as to seemingly exceed the capacity of LPS transporters (e.g., LptD) to translocate and assemble the LPS into a properly formed outer membrane. Inactivating *lptD* in A. baumannii did not result in periplasmic accumulation but did lead to toxic accumulation (backing up) of detergent-like lipid A pathway intermediates that interfered with growth, and similar to what we see here, growth was improved by chemical inhibition of LpxC (48). Therefore, we determined whether there might also be accumulation of lipid A pathway intermediates occurring in our K. pneumoniae *fabZ*/*lpxC* mutants. Analysis by LC-MS/MS showed that K. pneumoniae mutants harboring only a *fabZ* mutation accumulated a significant amount of LpxA product (Fig. 5). This would be consistent with redirection of substrate from fatty acid into the lipid A pathway and/or the compensatory downregulation of LpxC in *fabZ* mutants (36, 37, 40). The accumulation of LpxA product (LpxC substrate) measured here may however be consistent with the notion of increased abundance of substrate competing for binding to LpxC as a mechanism of resistance to LpxC inhibitors (36). To assess intermediate accumulation in the *fabZ/lpxC* mutant strain JWM0012, cells were cultivated initially in the presence of compound 2 and then dispensed into 96-well plates with twofold dilutions of compound 2, including wells containing no compound. Growth was monitored by optical density at 600 nm (OD_600_)_,_ and cells were harvested for LC-MS/MS analysis (Fig. 6). Cells subcultured without sufficient compound 2 showed clear accumulation of lipid X and disaccharide 1-monophosphate (DSMP) over time. This accumulation progressively decreased as cells were subcultured in increasing concentrations of compound 2. In the presence of higher concentrations of compound 2 (circa 4 to 8 µg/ml), lipid X and DSMP levels were similar to those of the wild-type strain. These results raise the possibility that toxic accumulation of lipid A pathway intermediates at the inner membrane might contribute to the inability of these mutants to grow without inhibition of LpxC.

### K. pneumoniae *fabZ*/*lpxC* mutants escape from inhibitor-dependent growth at high frequency.

The *lpxC*/*fabZ* mutations harbored by strain JWM0012 confer an inability to grow in the absence of an LpxC inhibitor. To examine the ability to escape from this extreme growth defect, cells were plated for mutant selection on solid medium lacking LpxC inhibitor. Mutants arose at a frequency of circa 6 × 10^−8^. A range of phenotypes were observed across several mutants picked for further study, indicating that different mechanisms of escape could be selected. Some mutants reverted to wild-type susceptibility to compound 2 (strains JRW0037 and JRW0039 [Table S1]), while others continued to demonstrate reduced susceptibility to compound 2 but did not require it for growth (JRW0040, JRW0041, and JRW0042 [Table S1]). Still others grew in the absence of compound 2 and in the presence of a high concentration of compound 2, but not with intermediate concentrations, similar to the eagle phenotype reported earlier (15) (JRW0035, JRW0036, and JRW0038 [Table S1]). Genome sequencing revealed that several of these mutants had acquired suppressor mutations in *lpxC* encoding the following amino acid substitutions in addition to the original V37L substitution: JRW0037 (L208P), JRW0038 (T6S), JRW0039 (P102L), and JRW0040 (T6S and D156Y) (Table S1). Western blot analysis of these mutants showed that the abundance of LpxC protein was still elevated relative to strains expressing wild-type LpxC (data not shown). This suggested that these changes, rather than restoring normal LpxC levels, functioned to reduce the catalytic efficiency of LpxC, effectively producing the same result as would a reduction in LpxC levels (or chemical inhibition); however, this remains to be confirmed. One mutant (JRW0041 [Table S1]) harbored a mutation in *lpxA* (encoding G201R) (Table S1). Although this change is not localized within the catalytic domain of LpxA based on the available published P. aeruginosa and E. coli LpxA structure (not shown), we suspect that this mutation somehow reduces LpxA activity, thereby reducing the flow of substrate into LpxC and allowing compound-independent growth. Intriguingly, this mutant had exceptionally high levels of LpxC protein (data not shown), suggesting a possible regulatory response to the lack of LpxC substrate being produced in this mutant. Last, we identified a mutation inactivating the heat shock stress response protein DnaK in one of the mutants (JRW0035 [Table S1]). The mechanism by which this mutation would restore compound 2-independent growth is not known; however, DnaK has been shown to be important in a variety of processes affecting survival under oxidative stress, nutrient limitation, and antibiotic exposure (49).

## DISCUSSION

Although rare, cases where bacteria have developed a dependence on an antibacterial compound for growth have been described, for example with streptomycin, colistin, and vancomycin (8, 50–55). Here we describe the isolation of K. pneumoniae mutants whose growth requires the inhibition of LpxC. Consistent with previous reports with other LpxC inhibitors (15, 36), individual mutations in the fatty acid biosynthetic gene *fabZ* and in the gene that encodes the target protein, *lpxC*, decreased susceptibility of K. pneumoniae to the LpxC inhibitor compound 2. However, K. pneumoniae mutants that encoded LpxC_V37G_ together with either FabZ_R121L_ or FabZ_F51L_ failed to grow in the absence of the LpxC inhibitor.

The LpxC variants identified here accumulated to much higher than normal levels in the mutant cells, possibly due to an apparent threefold increase of *lpxC* transcript abundance but possibly also due to increased protein stability. Overexpression of LpxC was a potential contributor to reducing compound susceptibility by a straightforward titration mechanism, as has been reported previously for P. aeruginosa (35). However, the increased levels of LpxC in conjunction with the *fabZ* mutations were of interest, as they prevented cell growth. Synthesis of LPS needs to be balanced with phospholipid (inner membrane) biosynthesis for formation of a functional cell envelope (37–39). Single *fabZ* mutations selected on LpxC inhibitors in E. coli may cause redirection of substrate from fatty acid synthesis into lipid A synthesis or simply decrease phospholipid synthesis, either of which might be expected to disturb this balance. It was recently shown that E. coli responds to *fabZ* mutations by reducing cellular levels of LpxC, presumably decreasing production of lipid A (37, 43, 44). However, the accumulation of LpxC_V37G_ in our mutants at higher than wild-type levels regardless of the presence or absence of a *fabZ* mutation suggested that the normal ability to adequately control LpxC levels had been lost. Presumably, this could occur during passaging in compound 2 because normal regulation was replaced by progressively increasing chemical inhibition of LpxC. The overly robust production of lipid A and severe accumulation of LPS in cells in the absence of the LpxC inhibitor represent an inability of these mutants to properly maintain membrane homeostasis and extend our understanding of the importance of this process to K. pneumoniae.

In addition to the overall membrane imbalance occurring in our mutants, there was an accumulation of lipid A pathway intermediates. Certain intermediates of the lipid A biosynthetic pathway (e.g., the substrate of LpxD) are detergent-like compounds, and it was suggested that if they should accumulate in cells, they would be toxic (56, 57). The idea of toxic accumulation has perhaps been best exemplified for biosynthetic pathways whose end products are not required for growth. An example is the biosynthesis of teichoic acid in Gram-positive bacteria. Genetic deletion of enzymes occurring early in the pathway is tolerated, but deletion of later steps is not, the so-called “essential gene paradox” (58). This has been attributed to toxic accumulation of pathway intermediates, although other explanations are possible. An essential gene paradox for lipid A biosynthesis was recently reported whereby LpxH and LpxK, which occur later in the biosynthetic pathway, were essential for *in vitro* growth of A. baumannii ATCC 19606 even though the earlier catalytic enzymes LpxA or LpxC were dispensable in that strain background (i.e., lipid A synthesis is not essential) (59, 60). Similar to our observations here, growth of A. baumannii ATCC 19606 downregulated for LpxH or LpxK was restored by an LpxC inhibitor (LpxC inhibitor-dependent growth), and this was associated with reduction of toxic intermediate accumulation. Deletion of the outer membrane LPS (referred to as lipooligosaccharide [LOS] in *A. baumannii*) transporter gene *lptD* in this strain reduced but did not eliminate cell-associated LOS and caused growth and permeability defects in cells, consistent with toxic accumulation, and these defects were relieved by inhibition of LpxC (48). The association of lipid A intermediates with growth inhibition described here may provide the first glimpse of a contribution of toxic intermediates to growth inhibition in K. pneumoniae, where lipid A synthesis is essential. This also provides further support to the notion that enzymes later in the lipid A pathway, such as LpxK, could have an added advantage of generating this type of toxic accumulation if inhibited and therefore be desirable therapeutic targets (44).

We do not know precisely how control of the cellular level of the LpxC variant selected here becomes dysregulated. One mechanism by which LpxC levels are controlled inside cells is via proteolytic degradation by the essential protease FtsH (61–64). An FtsH recognition motif was identified in the C terminus of LpxC (61, 62), and an additional site close to the LpxC N terminus may also be involved (62). A temperature-sensitive *ftsH* mutant accumulated LpxC upon shift to the nonpermissive temperature (65). More recently, it was proposed that a second protease may also play a role in controlling LpxC levels in E. coli (43). Since accumulation of LpxC in our mutants is mediated by amino acid substitutions located near the N terminus, one possibility is that these substitutions render LpxC refractory to proteolytic cleavage, but this remains to be experimentally shown. Accumulation of LpxC in the temperature-sensitive E. coli *ftsH* mutant also led to severe overproduction of membrane in the periplasm (65), similar to what we observed here for LpxC inhibitor-dependent mutants subcultured without compound 2. This suggests that accumulated LpxC alone can promote a significant overproduction of lipid A even in the absence of a mutation in *fabZ*, although the LpxC_V37l_ variant studied here did not show significant envelope abnormalities. The resulting membrane imbalance would be expected to affect the membrane permeability barrier and/or cause a fitness deficit. Consistent with this, *K. pneumoniae* strains harboring mutations only in *lpxC* (JWK0148-LpxC_V37G_ and JWM0107-LpxC_V37A_) grew well under our typical static experimental growth conditions but grew more slowly under shaking conditions, and growth was improved in the presence of compound 2. Growth was also poor on MacConkey agar, and sensitivity to rifampin was higher than for the wild-type strain, consistent with membrane defects (data not shown). This indicated LpxC_V37G_ or LpxC_V37A_ alone resulted in membrane imbalance, but not as severely as when combined with a *fabZ* mutation.

In conclusion, we exploited the availability of potent LpxC inhibitors to enable a K. pneumoniae serial passaging approach linking the selection of resistance to the generation of inhibitor-dependent growth. Individual *fabZ* mutations or mutations in the target gene *lpxC* decreased susceptibility to compound 2 as expected; however, the combination of these led to inhibitor-dependent growth. Underlying this was the idea that chemical inhibition of LpxC allowed mutants to emerge that had lost their biological mechanism(s) necessary to establish and maintain membrane homeostasis. This provides further support for the importance of the proper balancing of membrane synthesis and extends our understanding of this process to the important human pathogen K. pneumoniae. We also provide an intriguing view into the possibility of a contribution of toxic intermediate accumulation to growth inhibition in the context of an essential pathway, which may inform the selection of therapeutic targets within this pathway. Last, it is not known whether these mutants could emerge in the clinic during treatment with an LpxC inhibitor, but it is tempting to speculate that should this occur, the mutants may not be able to survive upon withdrawal of the drug.

## MATERIALS AND METHODS

### Bacterial strains, plasmids, and growth conditions.

The bacterial strains and plasmids used in this study are listed in Table S1 in the supplemental material. K. pneumoniae ATCC 43816 and E. coli ATCC 25922 were purchased from the American Type Culture Collection (ATCC). Cells were routinely grown in cation-adjusted Mueller-Hinton broth (MHB) (3.0 g/liter beef extract, 17.5 g/liter acid hydrolysate of casein, 1.5 g/liter starch, 20 to 25 mg/liter calcium, 10 to 12.5 mg/liter magnesium) or lysogeny broth (LB) (10 g/liter tryptone, 5 g/liter yeast extract, and 10 g/liter NaCl). Media were supplemented with carbenicillin (500 µg/ml for K. pneumoniae) for mutant construction by recombineering (66). The LpxC inhibitor, compound 2 (14), was synthesized at Novartis.

### Determination of MICs and serial passage for isolating mutants resistant to LpxC inhibitor compound 2.

For MIC determinations, bacterial strains were streaked on MHB agar or MHB agar supplemented with compound 2 as indicated and grown overnight. LpxC inhibitor compound 2 was twofold serially diluted in the 96-well round-bottom plate in 50 µl of MHB or LB. Bacterial suspensions were prepared from overnight colonies using BBL prompt (BD Biosciences, San Jose, CA) according to the supplied instructions and used to introduce approximately 5× 10^5^ colony-forming units (CFU) in 50 µl in each well. Plates were incubated at 37°C, and bacterial growth was determined by monitoring optical density at 600 nm (OD_600_).

Serial passage experiments were based on the 96-well plate MIC format described above. From the MIC determination, 2 µl of culture (50× dilution) from wells at one compound 2 dilution step below the MIC (i.e., the highest concentration of compound 2 where the cells grew) was used to inoculate a second plate set up similarly to the first plate. This process was repeated for a total of 12 passages, and the MIC of the culture progressively increased. Culture from a passage that starts to show compound 2-dependent growth was streaked on MHB agar containing compound 2 to isolate individual mutants. Individual mutants were then tested with the MIC assay again to ensure they retain the growth-dependent phenotype.

### Whole-genome sequencing of isolated mutants.

Genomic DNA was prepared from bacterial cells using the Qiagen DNeasy Blood and Tissue kit (Qiagen, Redwood City, CA) according to the manufacturer’s instructions, except that samples were eluted in and then diluted to 8 ng/μl with nuclease-free water. Whole-genome sequencing and identification of mutations were performed according to standard protocols.

### Construction of mutants JWK0148 (LpxC_V37G_), JWK0150 (LpxC_V37G_/FabZ_R121L_), and JWK0151 (LpxC_V37G_/FabZ_F51L_) in K. pneumoniae ATCC 43816.

Plasmids and oligonucleotides used in this study are listed in Tables S1 and S2. K. pneumoniae ATCC 43816 was transformed with plasmid pTU430 (Table S1), which harbors the recombinase from pKD46 (66), for chromosomal engineering and the *sacB* gene for counterselection. DNA substrates encoding amino acid substitutions for LpxC_V37G_, FabZ_R121L_, and FabZ_F51L_ were PCR amplified from mutant strain JWM0012 or JWM0013 using primer pairs Kp.lpxCF/Kp.lpxCR or Kp.fabZF/Kp.fabZR and gel purified. K. pneumoniae ATCC 43816 harboring the recombinase plasmid pTU430 was grown overnight in LB with carbenicillin (500 µg/ml) and diluted 1:100 in fresh LB with carbenicillin (150 µg/ml) and 0.2% arabinose and grown to an OD_600_ of ∼0.6. Cells were pelleted by centrifugation and washed twice in ice cold 10% glycerol. The linear DNA substrates encoding mutations were then introduced to cells by electroporation. Transformants were plated on LB plates with compound 2 at 4 µg/ml. Candidate mutants were cured of plasmid pTU430 by plating on LB agar with 8% sucrose and lacking NaCl. A PCR-confirmed mutant harboring the chromosomal mutation encoding LpxC_G37V_ was designated JWK0148. Mutations encoding FabZ_R121L_ and FabZ_F51L_ were then introduced into the genome of strain JWK0148 by a second round of recombineering. PCR-confirmed mutants designated JWK0150 and JWK0151 had mutations encoding LpxC_V37G_/FabZ_R121L_ and LpxC_V37G_/FabZ_F51L_, respectively.

10.1128/mSphere.00508-18.5 TABLE S2 Primers used in this study. Download Table S2, PDF file, 0.04 MB. Copyright © 2018 Mostafavi et al. 2018 Mostafavi et al. This content is distributed under the terms of the Creative Commons Attribution 4.0 International license.

### Western blot for detection of LpxC proteins.

Bacterial strains were grown overnight in 5 ml LB (K. pneumoniae ATCC 43816 and *lpxC* mutant JWK0148) or LB supplemented with 32 µg/ml compound (*fabZ*/*lpxC* mutants JWM0012 and JWK0150) at 37°C with shaking. The cultures were diluted 1:20 the next day in 30 ml LB alone or supplemented with 32 µg/ml compound 2 and incubated again in the 37°C shaker. In either the presence or absence of compound 2, *lpxC* or *fabZ*/*lpxC* mutants showed a significantly higher LpxC abundance compared to the wild type. For simplification, we showed only the results for strains grown with 32 µg/ml compound 2, in which wild-type culture was significantly suppressed. (In comparison, when grown in the absence of compound 2, growth of *fabZ*/*lpxC* mutants JWM0012 or JWK0150 was significantly suppressed.) Culture density was monitored by OD_600_ every half hour, and cells were collected at 2 h. The culture was normalized to an OD_600_ of 0.5, pelleted, and suspended in 1:2 Tricine sample buffer, sonicated for 1 min, and boiled at 70°C for 20 min. Samples were loaded and run on a Bolt 4 to 12% Bis-Tris Plus gel (catalog no.NW04127BOX; Thermo Fisher Scientific, Waltham, MA) with MES buffer (50 mM MES, 50 mM Tris, 1 mM ETDA, 0.1% SDS). Proteins were then transferred to a nitrocellulose membrane using an iBlot system (Thermo Fisher Scientific, Waltham, MA) according to the supplied instructions. LpxC protein was detected using an iBind Flex Western device (Thermo Fisher Scientific, Waltham, MA) and visualized with the Odyssey CLX imaging system (LI-COR Biosciences, Lincoln, NE).

### Liquid chromatography coupled to tandem mass spectrometry (LC-MS/MS) detection of lipid A pathway intermediates.

#### (i) Sample preparation.

K. pneumoniae ATCC 43816, *fabZ* mutant JWM0009, or *fabZ*/*lpxC* JWM0012 was grown overnight in MHB or MHB supplemented with 32 µg/ml of compound 2 (for strain JWM0012). The cultures were then diluted to an OD_600_ of ∼0.05 in fresh MHB medium and grown for 1 h in a flask with shaking at 37°C to an OD_600_ of ∼0.15 to 0.2. An 11 point 1:2 serial dilution of compound 2 was made in DMSO at 100×, and 10 µl was stamped into 96-well 2-ml deep-well plates for a final assay concentration series starting at 128 µg/ml. DMSO was also stamped at a 10-µl volume for the no-compound control. One plate was used for each time point, and each serial dilution of the compound was replicated twice. Cells were added to the 96-well plates at a volume of 1 ml, and then plates were sealed with a breathable seal and incubated at 37°C with shaking. After each time point, the plate was removed from the incubator, and 100 µl of the cell solution was transferred to a clear 96-well plate for OD_600_ reading. To the remaining 900 µl of cell culture, 1 ml of Genlantis SoluLyse detergent lysis buffer was added, and the C10 P. aeruginosa LpxA product (Alberta Research Chemicals, Edmonton, Canada) internal standard was spiked in at a final concentration of 1 nM. Plates were shaken at room temperature for 30 min before being spun at 3,000 rpm for 15 min to pellet cellular debris. Solid-phase extraction was performed using Waters Oasis WAX SPE 96-well plates (10 mg sorbent per well, 30-µm particle) on a vacuum manifold. Solid-phase extraction products (SPE) were conditioned with 500 µl of methanol and reequilibrated with 500 µl of water. From the pelleted cell lysate, two 900-µl portions of supernatant were transferred to SPE, with flowthrough being discarded. SPE were washed with 500 µl of 2% formic acid, followed by 500 µl of acetonitrile. LPS intermediates were eluted from SPE with 75 µl of 5% ammonium hydroxide in methanol into a 96-well polypropylene plate containing 15 µl of 10% formic acid in methanol per well to acidify the samples to approximately pH 5 to 6. Note that allowing the LPS biosynthetic intermediates to incubate in basic solution will result in poor results due to hydrolysis.

#### (ii) Detection of LPS intermediates by normal-phase LC-MS/MS.

Multiple reaction monitoring (MRM) data were acquired on a Sciex 4000 QTRAP mass spectrometer with Turbo V ion source coupled to an Agilent 1100 LC. Both systems are well-suited to normal-phase (NP) operation when the appropriate glass and steel capillaries are used, as well as appropriate pump seals. Other LC-MS systems may be less suitable for the buffers employed, consult the vendor. Mobile phase A consisted of 80% chloroform, 19.5% methanol, and 0.5% ammonium hydroxide. Mobile phase B consisted of 45% chloroform, 45% methanol, 9.5% water, and 0.5% ammonium hydroxide. Flow was applied at 200 µl/min to a Waters Acquity UPLC BEH HILIC column (130-Å pore; 1.7-µm particle) (2.1 by 100 mm). Samples were kept at 4°C in the autosampler prior to injecting 10 µl onto the column. Samples were loaded in 10% mobile phase B, and this composition was held for the first minute of chromatography. Mobile phase B was increased to 70% at 3 min and to 99% at 6 min, held at 99% until 9 min, and then reduced to 10% at 9.1 min. The system was reequilibrated for 4 min between sample injections. MS/MS data were collected between minutes 2 to 11. The mass spectrometer source heater was set at 450°C, IonSpray voltage of −4,500 V, curtain gas of10 liters/min, collision gas of 8 liters/min, nebulizer gas of 35 liters/min, and heater gas of 40 liters/min. MRM settings are shown in Table S3. MRM data were acquired with a duty cycle of 1.26 s per scan. All transitions were tuned on samples of authentic standards in infusion mode prepared as described previously (59). Integrated retention times were matched to authentic standard elution profiles as well. Peaks were integrated using Skyline software and then normalized to OD_600_ and internal standard for each monitored metabolite. For Fig. 5, results are shown for the 8-h time point and are the average of eight replicates. Figure 6 shows the time course experiment, with results from duplicates at the 1-, 3-, 5-, and 8-h time points.

10.1128/mSphere.00508-18.6 TABLE S3 MRM settings for monitoring K. pneumoniae LPS intermediates and P. aeruginosa internal standard. Download Table S3, PDF file, 0.1 MB. Copyright © 2018 Mostafavi et al. 2018 Mostafavi et al. This content is distributed under the terms of the Creative Commons Attribution 4.0 International license.
