# Supplementary material for: Interplay of Klebsiella pneumoniae fabZ and lpxC Mutations Leads to LpxC Inhibitor-Dependent Growth Resulting from Loss of Membrane Homeostasis
Source: mSphere. 2018 Oct 31;3(5):e00508-18. doi: 10.1128/mSphere.00508-18 (PMC6211225; doi:10.1128/mSphere.00508-18)
Supplement: TABLE S1 [file sph006182676st1.pdf]

Strain	Relevant characteristics	Reference or source
K. pneumoniae		
ATCC43816	Wild type strain	American Type Culture Collection (ATCC)
JWM0009	LpxC inhibitor resistant mutant derived from ATCC 43816; $FabZ_{R121H}$	This study
JWM0010	LpxC inhibitor resistant mutant derived from ATCC 43816; $FabZ_{A69V}$	This study
JWM0011	LpxC inhibitor resistant mutant derived from ATCC 43816; $FabZ_{R126C}$	This study
JWM0012	Derived from ATCC 43816 via serial passage in LpxC inhibitor compound 2, $LpxC_{V37G,}$ $FabZ_{R121L}$; additional mutations: insertion TTTCGCTA into RamR caused frameshift from T50	This study
JWM0013	Derived from ATCC 43816 via serial passage in LpxC inhibitor compound 2, $LpxC_{V37G}$ $FabZ_{F51L}$; additional mutations: $AsnS_{P252S}$, $NsrR_{R69RNGSIHLGR}$	This study
JWM0107	LpxC inhibitor resistant mutant derived from ATCC 43816; $LpxC_{V37A}$	This study
JWK0148	Engineered derivative of ATCC 43816; $LpxC_{V37G}$	This study
JWK0150	Engineered derivative of ATCC 43816; $LpxC_{V37G}$ $FabZ_{R121L}$	This study
JWK0151	Engineered derivative of ATCC 43816; $LpxC_{V37G}$ $FabZ_{F51L}$	This study
JRW0035	Derived from JWM0012, does not require compound 2 to grow, eagle phenotype, $LpxC_{V37G}$ $FabZ_{F51L}$ $DnaK_{N44\ to\ stop}$	This study
JRW0036	Derived from JWM0012, does not require compound 2 to grow, eagle phenotype, $LpxC_{V37G}$ $FabZ_{F51L}$, no suppressor mutations in *lpxC* or *fabZ* (not selected for deep sequencing).	This study
JRW0037	Derived from JWM0012, does not require compound 2 to grow, susceptibility to compound 2 restored to that of WT ATCC43816, $LpxC_{V37G}$ $FabZ_{F51L}$ $LpxC_{L208P}$	This study
JRW0038	Derived from JWM0012 and does not require compound 2 to grow, eagle phenotype, $LpxC_{V37G}$ $FabZ_{F51L}$ $LpxC_{T6S}$	This study
JRW0039	Derived from JWM0012, does not require compound 2 to grow, susceptibility to compound 2 restored to that of WT ATCC43816, $LpxC_{V37G}$ $FabZ_{F51L}$ $LpxC_{P102L}$	This study
JRW0040	Derived from JWM0012, does not require compound 2 to grow, highly resistant to compound 2, $LpxC_{V37G}$ $FabZ_{F51L}$ $LpxC_{T6S,\ D156Y}$	This study
JRW0041	Derived from JWM0012, does not require compound 2 to grow, highly resistant to compound 2, $LpxC_{V37G}$ $FabZ_{F51L}$ $LpxA_{G201R}$	This study
JRW0042	Derived from JWM0012, does not require compound 2 to grow, highly resistant to compound 2, $LpxC_{V37G}$ $FabZ_{F51L}$, no suppressor mutations in *lpxC* or *fabZ* (not selected for deep sequencing).	This study
E. coli		
ATCC 25922	Wild type strain, recommended reference strain for antimicrobial susceptibility tests	ATCC
JWM0103	$LpxC_{V37G}$, derived from ATCC 25922 via serial passage in LpxC inhibitor	This study

JWM0102	$FabZ_{A69V}$ $LpxC_{G36R}$, derived from ATCC 25922 via serial passage in LpxC inhibitor compound 2	This study
JRW0005	BW25113 *ΔtolC* harbored pMMB206-EcLpxC plasmid	This study

Plasmid		
pTU430	Derived from pFLP2 (1), *sacB*, ori1600, encodes lambda Red genes (*exo*, *bet*, *gam*) from pKD46; Ap^r	This study
pMMB206-Ec LpxC	*E. coli lpxC* under IPTG induction in pMMB206	This study

1. **Hoang TT, Karkhoff-Schweizer RR, Kutchma AJ, Schweizer HP.** 1998. A broad-host-range Flp-FRT recombination system for site-specific excision of chromosomally-located DNA sequences: application for isolation of unmarked Pseudomonas aeruginosa mutants. Gene **212:**77-86.
