# Supplementary figures and images for: Interplay of Klebsiella pneumoniae fabZ and lpxC Mutations Leads to LpxC Inhibitor-Dependent Growth Resulting from Loss of Membrane Homeostasis
Source: mSphere. 2018 Oct 31;3(5):e00508-18. doi: 10.1128/mSphere.00508-18 (PMC6211225; doi:10.1128/mSphere.00508-18)

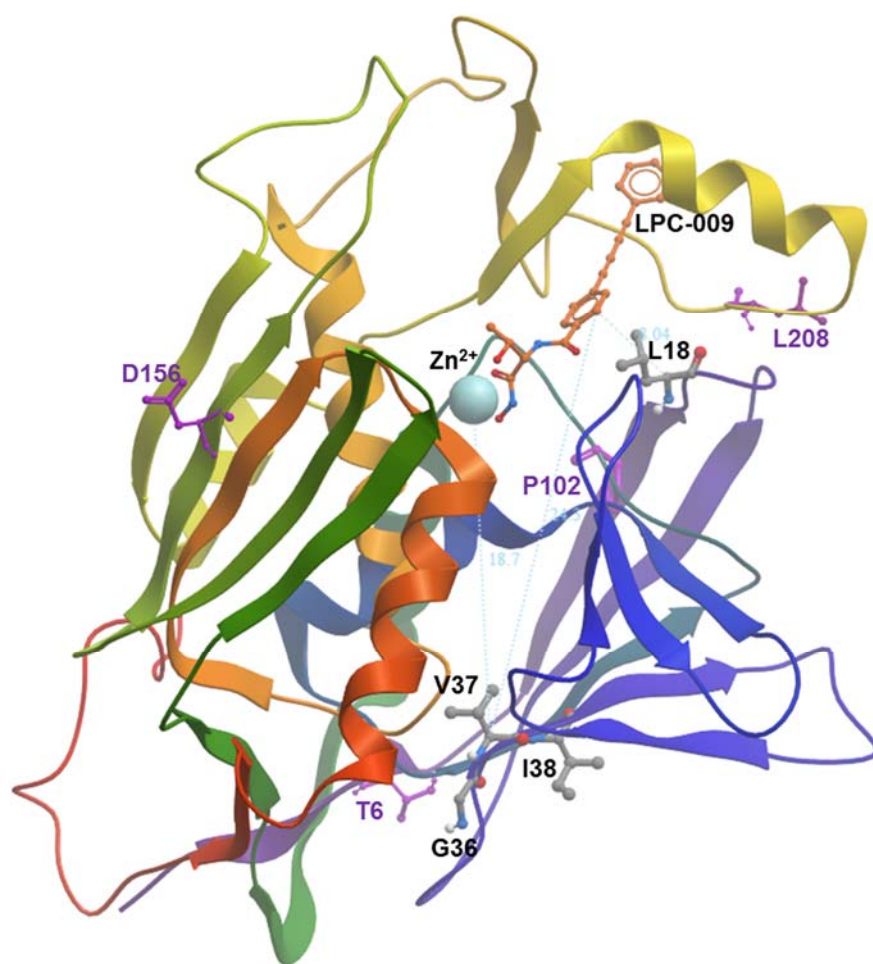

**Fig. S1**

Supplement: FIG S1 [file sph006182676sf1.pdf]

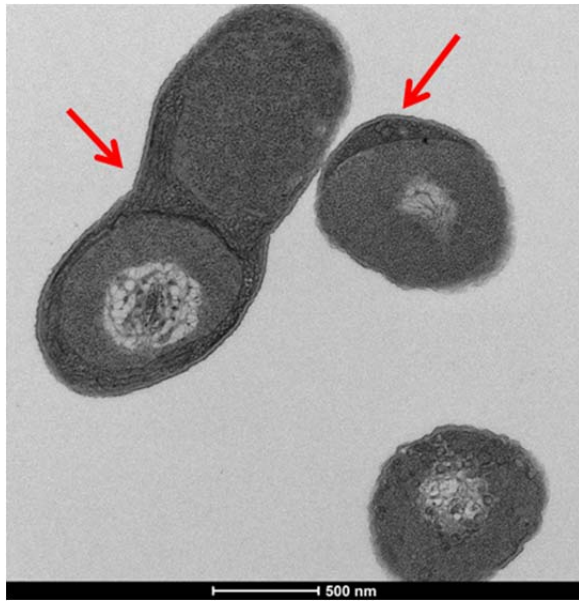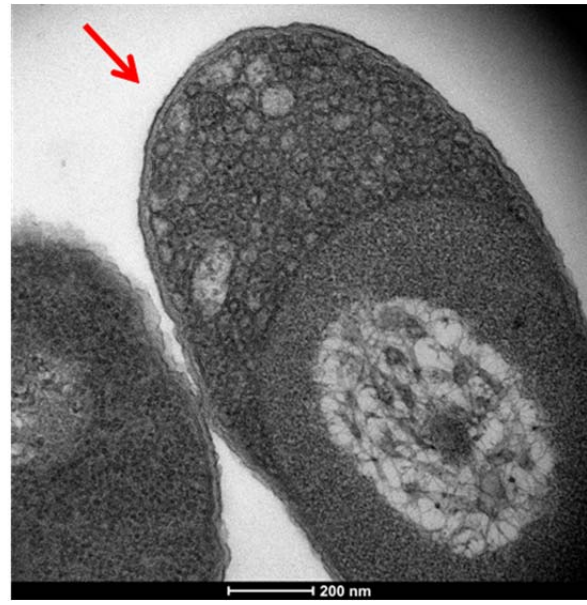

**Figure S2**

Supplement: FIG S2 [file sph006182676sf2.pdf]

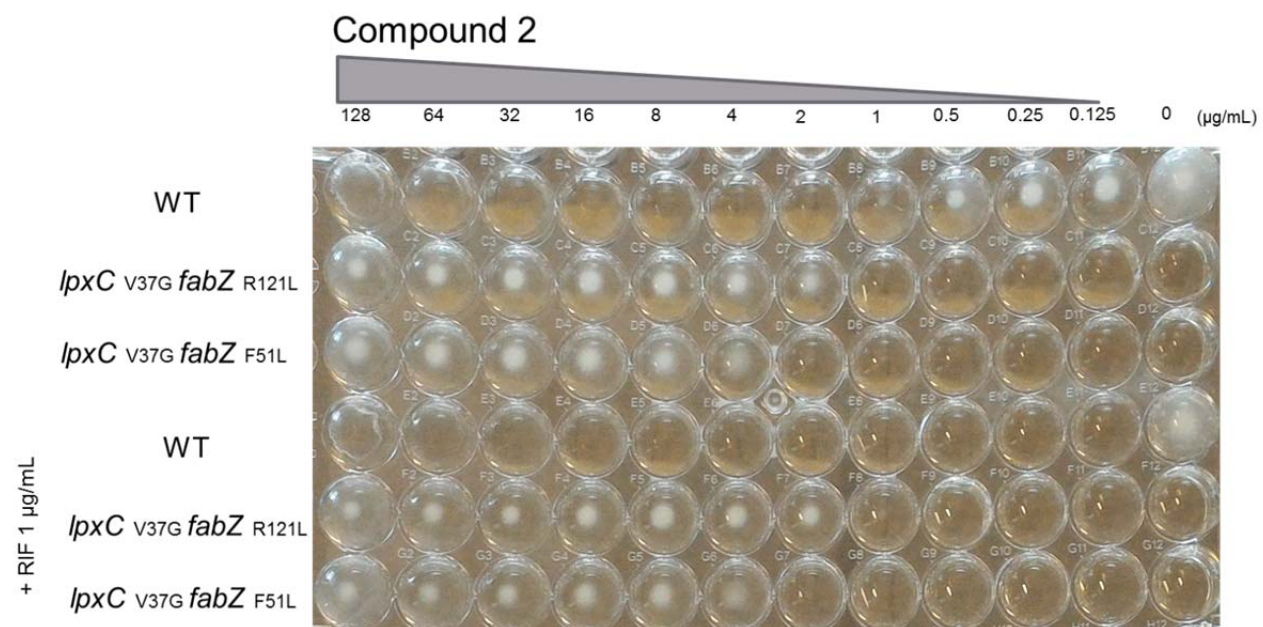

**Fig. S3**

Supplement: FIG S3 [file sph006182676sf3.pdf]
