# Supplementary material for: Interplay of Klebsiella pneumoniae fabZ and lpxC Mutations Leads to LpxC Inhibitor-Dependent Growth Resulting from Loss of Membrane Homeostasis
Source: mSphere. 2018 Oct 31;3(5):e00508-18. doi: 10.1128/mSphere.00508-18 (PMC6211225; doi:10.1128/mSphere.00508-18)
Supplement: TABLE S2 [file sph006182676st2.pdf]

Primer name	Sequence 5’ to 3’
Kp.fabZ F	GTG CTG GGC GAT AAC GTA GTT ATC
Kp.fabZ R	CGT TTC AGC CCT TCG ATG TTA ACG
Kp.lpxC F	CGG CTG AAA TGG CTA TCT CTT CTC
Kp.lpxC R	GAG ATG ACG AAT AAC GGT GCG AAT G
Ec fabZ F	TGA TGA CAT GAG CAA GCG TCT
Ec fabZ R	TTC GAC ATG GGG TCC AAC GAT AC
Ec lpxC F	CGG TTG GAT AGG TAA TTT GGC GAG
Ec lpxC R	GGA TGG TTA AAA TCG ATG GTG AAA
