# Supplementary material for: Interplay of Klebsiella pneumoniae fabZ and lpxC Mutations Leads to LpxC Inhibitor-Dependent Growth Resulting from Loss of Membrane Homeostasis
Source: mSphere. 2018 Oct 31;3(5):e00508-18. doi: 10.1128/mSphere.00508-18 (PMC6211225; doi:10.1128/mSphere.00508-18)
Supplement: TABLE S3 [file sph006182676st3.pdf]

Analyte	Q1 (*m/z*)	Q1 (res)	Q3 (*m/z*)	Q3 (res)	Dwell (ms)	DP (V)	EP (V)	CE (V)	CXP (V)
UDP-3-O-[(R)-3-OH-C_{14}]-GlcNAc	832.3	Unit	158.9	Unit	40	-100	-10	-80	-12
UDP-3-O-[(R)-3-OH-C_{14}]-GlcNAc	832.3	Unit	273	Unit	40	-100	-10	-70	-10
UDP-3-O-[(R)-3-OH-C_{14}]-GlcNAc	832.3	Unit	385	Unit	40	-100	-10	-50	-10
UDP-3-O-[(R)-3-OH-C_{14}]-GlcN	790.3	Unit	158.9	Unit	40	-90	-10	-85	-12
UDP-3-O-[(R)-3-OH-C_{14}]-GlcN	790.3	Unit	273	Unit	40	-90	-10	-65	-10
UDP-3-O-[(R)-3-OH-C_{14}]-GlcN	790.3	Unit	385	Unit	40	-90	-10	-52	-10
UDP-3-O-[(R)-3-OH-C_{14}]-GlcN	790.3	Unit	546.2	Unit	40	-90	-10	-44	-5
UDP-2,3-diacyl-GlcN	1016.5	Unit	158.9	Unit	40	-120	-10	-108	-10
UDP-2,3-diacyl-GlcN	1016.5	Unit	273	Unit	40	-120	-10	-74	-10
UDP-2,3-diacyl-GlcN	1016.5	Unit	385	Unit	40	-120	-10	-60	-10
Lipid X	710.4	Unit	466.2	Unit	40	-80	-10	-50	-10
Lipid X	710.4	Unit	240.1	Unit	40	-80	-10	-73	-10
DSMP	1323.9	Unit	79	Unit	40	-120	-10	-130	-10
DSMP	1323.9	Unit	1079.7	Unit	40	-120	-10	-90	-12
DSMP	1323.9	Unit	835.5	Unit	40	-120	-10	-80	-5
DSMP	1323.9	Unit	895.5	Unit	40	-120	-10	-80	-5
DSMP	1323.9	Unit	651.3	Unit	40	-120	-10	-110	-5
Lipid IV_A	701.4	Unit	243.2	Unit	40	-110	-10	-45	-15
Lipid IV_A	701.4	Unit	79	Unit	40	-110	-10	-130	-5
Lipid IV_A	701.4	Unit	588.3	Unit	40	-110	-10	-35	-15
Lipid IV_A	701.4	Unit	466.2	Unit	40	-110	-10	-40	-15
Lipid IV_A	701.4	Unit	1079.7	Unit	40	-110	-10	-35	-15
UDP GlcNAc	606.4	Unit	385	Unit	40	-80	-10	-37	-5
UDP GlcNAc	606.4	Unit	282.4	Unit	40	-98	-10	-42	-11
UDP GlcNAc	606.4	Unit	273	Unit	40	-91	-10	-46	-14
UDP-3-O-[(R)-3-OH-C_{10}]-GlcNAc	776.3	Unit	158.9	Unit	40	-118	-10	-80	-5
UDP-3-O-[(R)-3-OH-C_{10}]-GlcNAc	776.3	Unit	273	Unit	40	-118	-10	-60	-5
UDP-3-O-[(R)-3-OH-C_{10}]-GlcNAc	776.3	Unit	385	Unit	40	-118	-10	-45	-5
